# Beclin1 inhibition promotes autophagy and decreases gemcitabine–induced apoptosis in Miapaca2 pancreatic cancer cells

**DOI:** 10.1186/1475-2867-13-26

**Published:** 2013-03-13

**Authors:** Xiaoshu Li, Jun Yan, Lisheng Wang, Fengjun Xiao, Yuefeng Yang, Xiaozhong Guo, Hua Wang

**Affiliations:** 1 Department of Gastroenterology, Shenyang General Hospital of PLA, 83 Wenhua Road, Shenyang, 110016, P.R. China; 2 Department of Gastroenterology, The Fourth People’s Hospital of Shenyang, 20 Huanghe South Street, Shenyang, 110031, P.R China; 3 General Hospital of PLA, 28 FuXing Road, Beijing, 100853, P.R. China; 4 Department of Experimental Hematology, Beijing Institute of Radiation Medicine, 27 TaiPing Road, Beijing, 100850, P.R. China

## Abstract

**Background:**

Beclin1 is a well-known key regulator of autophagy, which is also a haploinsufficient tumor suppressor. Current studies revealed that down-regulation or monoallelic deletions of Beclin1 were frequently found in various cancers. The purpose of this study was to investigate the effects of Beclin1 inhibition on autophagy and Gemcitabine-induced apoptosis of pancreatic cancer cells.

**Methods:**

Beclin1 expression was inhibited by siRNA transduction and gene expression was determined by Real-time PCR and Western blot. The effects of Beclin1 inhibition on autophagy and Gemcitabine-induced apoptosis of Miapaca2 cells were analyed through LC3 expression, cell viability, cell cycle and apoptosis by using Western blot.

**Results:**

We observed that Beclin1 silence promoted microtubule-associated protein 1 light chain 3-II (LC3-II) protein formation and increased punctate fluorescent signals in Miapaca2 cells transfected with green fluorescent protein (GFP)-tagged LC3. Beclin1 inhibition showed a greater suppressive effect on Gemcitabine-induced apoptosis of Miapaca2 cells.

**Conclusion:**

Our data suggested that Beclin1 silence not only up-adjusted autophagy process, but also played an important role in the regulation of apoptosis. Beclin1 inhibition could inhibit apoptosis signaling induced by Gemcitabine in Miapaca2 cells.

## Background

Autophagy is an evolutionary conserved process in which cell engulfs cytoplasmic constituents within a double-membrane vacuole (termed autophagosome) and delivers them to the lysosome for degradation. Autophagy contributes to maintain cellular homeostasis as a result of quality control of both proteins and organelles [1-3]. In addition to its basic role in the turnover of proteins and organelles, autophagy has multiple physiological and pathophysiological functions including supply of nutrients and energy for cell survival and protection of cells against environmental stress [4,5]. Some evidences demonstrate that increased numbers of autophagosomes have been associated with forms of non-apoptotic cell death [6,7], that is distinct from apoptosis, a form of cell suicide mediated by caspase activation. Autophagy plays an important role in the modulation of cancer development and in the determination the response of many cancer cells to pro-apoptotic-related chemotherapy. The functional relationship between apoptosis and autophagy is complicated.

Beclin1 is a phylogenetically conserved protein that is essential for autophagy. In fact, the first association between autophagy and cancer was the landmark discovery of Beclin1, which is also a haploinsufficient tumor suppressor [1]. Current studies revealed that down-regulation or monoallelic deletions of Beclin1 were frequently found in various cancers including human breast carcinoma, oophoroma, prostatic carcinoma and others [8-10]. Accordingly the expression of Beclin1 may play an important role in tumorigenesis.

The therapeutic goal of cancer treatment is to trigger tumor-selective cell death. Pancreatic cancer, a highly lethal disease, is usually diagnosed at an advanced stage for which there is little or no effective therapy. Gemcitabine, an anti-cancer chemotherapeutic drug which affects cell cycle and causes apoptosis, is an important option in curing or controlling various tumors including pancreatic cancer. Gemcitabine offers both symptomatic and survival benefits for pancreatic carcinoma. Emerging evidences have shown that autophagy plays an important role in the regulation of chemosensitivity to anti-cancer drugs and affects proliferation in some cancer cells. However, the role of Beclin1 in regulation of Gemcitabine-induced autophagy and apoptosis remain unclear.

In this study,we examined the autophagy of human pancreatic cancer Miapaca2 cells transduced with Beclinl-siRNA. Meanwhile the relationship between Beclinl inhibition and Gemcitabine induced apoptosis were detected in Miapaca2 cells.

## Methods

### Cell lines and cell culture

The human pancreatic cancer Miapaca2 cells were obtained from Beijing Institute of Radiation Medicine. Cells were cultured with Dulbecco’s modified Eagle’s medium (DMEM; Sigma, St. Louis, MO, USA) supplemented with 10% fetal bovine serum (FBS; Hyclone, USA). Cultures were incubated in a humidified atmosphere of 95% air and 5% CO_2_ at 37°C.

### RNA interference of Beclin1

Miapaca2 cells were seeded in 24-well plates (1.5 × 10^5^ cells per well) and incubated overnight. A negative control random small interfering RNA (NC-siRNA) or Beclin1-targeted siRNA (1 μg/well) was transfected by using Lipofectamine 2000 (Invitrogen, Carlsbad, CA, USA) according to the manufacturer’s protocol. After 48 h transfection, cells were collected and cell lysates were subjected to immunoblotting of Beclin1. Beclin1-siRNA, NC-siRNA and FAM-siRNA were provided by Shanghai Genepharma Co., Ltd. The negative control sequence was GTTCTCCGAACGTGTCACGT. The interfering sequence of Beclin1 was GCTGCCGTTATACTGTTCT.

### Western blot analysis of Beclin1

After transfection, the Miapaca2 cells were washed twice with PBS, and lysed with a solution containing Tris–HCl (50 mmol/L, pH 6.8), SDS (10% w/v), glycerol (10%), and dithiothreitol (10 mmol/L), supplemented with protease inhibitor mix. Cell lysates were centrifuged at 12,000 *g* for 20 min. Equal amounts of the protein were suspended in 5×Laemmli buffer and resolved by SDS–PAGE and transferred onto a PVDF membrane. After blocking with 5% non-fat dry milk in TBS for 1 h at room temperature, membranes were washed 3 times and incubated for 1 h with anti-Beclin1 antibody (Sinopharm chemical Reagent Co. Ltd, China) at room temperature then at 4°C overnight. Then the PVDF membrane was treated with a horseradish peroxidase-conjugated anti-rabbit IgG antibody (Santa Cruz Biotechnology, CA, USA) for 45 minutes at room temperature. Specific bands were detected by an enhanced chemiluminescence system (Pierce, Rockford, IL, USA). Anti-β-Actin (Santa Cruz Biotechnology, CA, USA) was used to ensure equal loading. Band intensity was semi-quantified using Scion Image software after conversion to digitalizing image using an image scanner (GT9700F; Epson, Tokyo, Japan).

### Real-time analysis of Beclin1 gene

The primers were designed by Primer premier 5.0 software and synthesized by Shanghai Invitrogen Co. Ltd. China. Beclin1 forward sequence (5^′^to3^′^): GGTGTCTCTCGCAGATTCATC; Beclin1 reverse sequence (5^′^to3^′^): TCAGTCTTCGGCTGAGGTTCT. Total RNA was prepared from Miapaca2 cells transfected with Beclin1-siRNA or NC-siRNA according to the manufacturer’s instructions and the concentration of total RNA was determined.The RNA was then reversely transcribed in a 20 μL reaction mixture according to the instructions of RevertAid First strand cDNA syntherio Kit (Fermentas, China).The PCR program for Beclin1 and GAPDH (Santa Cruz Biotechnology, CA, USA) included one cycle at 95°C for 2 min, 39 cycles at 95°C for 20 s,65°C for 20 s, 72°C for 30 s, and finally extension at 72°C for 10 min, the PCR products of Beclin1 gene and internal controls were detected by electrophoresis in agrose gel.

### Western blot analysis of LC3-α

After transfection of Beclin1-siRNA or NC-siRNA for 48 h, the Miapaca2 cells were treated as previous process described to detect expression of LC3-;α protein with anti-LC3 antibody (MBL International) and anti-rabbit IgG antibody (Santa Cruz Biotechnology, CA, USA).

### Involvement of LC3

PEGFP-LC3 plasmid was kindly gifted by professor Noboru Mizushima and Tamotsu Yoshimori (Tokyo Dentistry University). Miapaca2 cells were plated in 24-well plates (1.5 × 10^5^ cells/well) and transfected with PEGFP-LC3 plasmid and Beclin1-siRNA or NC-siRNA plasmid by using the Lipofectamine 2000. After 48 h transfection, cells were washed with PBS and permeabilized with 0.1% TritonX-100 for 10 min, staining with DAPI (1:10 dilution with buffer fluid) for 10 min. Then autophagosome in cells were observed through green fluorescent protein (GFP) in cells by Confocal laser scanning microscope (Zeiss LSM510, German).

### Cell viability measurement

Cell viability was deteced by Cell Counting Kit-8 (CCK8, Dojindo Molecular Technologies, Kumamoto, Japan). After transfection with Beclin1-siRNA or NC-siRNA for 24 h, Miapaca2 cells were seeded in 96-well plates at a density of 5×10^3^ cells/well and incubated, Untreated Miapaca2 cells were as a control group.10 μL CCK8 was put into each well after transfection for 30 h, 48 h, 72 h and 96 h, then the cells were incubated in a humidified 5% CO_2_ atmosphere at 37°C for 1 h. The absorbance of the solution was read spectrophotometrically at 450 nm through a microtitre plate reader (Thermo Varioskan flash, USA). Cell viability was calculated according to the following formula: cell viability (%) = A450 (tested group - blank group)/A450 (control group - blank group) × 100%. Three replicates were performed for each treatment.

### Analysis of the cell cycle

Miapaca2 cells were transfected with corresponding plasmids in 24-well plates and incubated for 24 h. Then cells were exposed to adequate density of Gemcitabine (3 μg/ml). After 24 h of treatment, cells were harvested by trypsinization, washed twice in cold PBS. Then fixed with cold 70% ethanol, and stored at 4°C until analyzed. The cells were resuspended in PBS added 2 μL of RNaseA (10 mg/mL), Propidium iodide (final concentration, 50 μg/mL; BD,USA), then incubated at 4°C in the dark for 20 min, and DNA content was detected by flow cytometry (BD FACSCalibur, USA). The relative proportions of cells in the G_2_/G_1_and S phases of the cell cycle were determined from the flow cytometry data.

### Apoptosis detection by flow cytometry

Miapaca2 cells were transduced with corresponding plasmids in 24-well plates and incubated for 24 h. Then cells were exposed to adequate density of Gemcitabine (3 μg/ml). After 24 h of treatment, cells were harvested by trypsinization, washed twice with cold PBS. After staining with the combination of AnnexinV/fluorescein isothiocyanate (FITC) and propidium iodide (PI) (Annexin-V: FITC Apoptosis Detection Kit, BD Pharmingen), the cells were immediately analyzed by flow cytometry.

### Statistical analyses

All statistical analyses were performed using SPSS11.5 software. The results expressed as means ± SD and were compared using Student’s *t* test to determine statistical significance between control and test groups. p < 0.05 was considered statistically significant.

## Results

### SiRNA inhibition of Beclin1 expression by Miapaca2 cells

**Figure 1:**
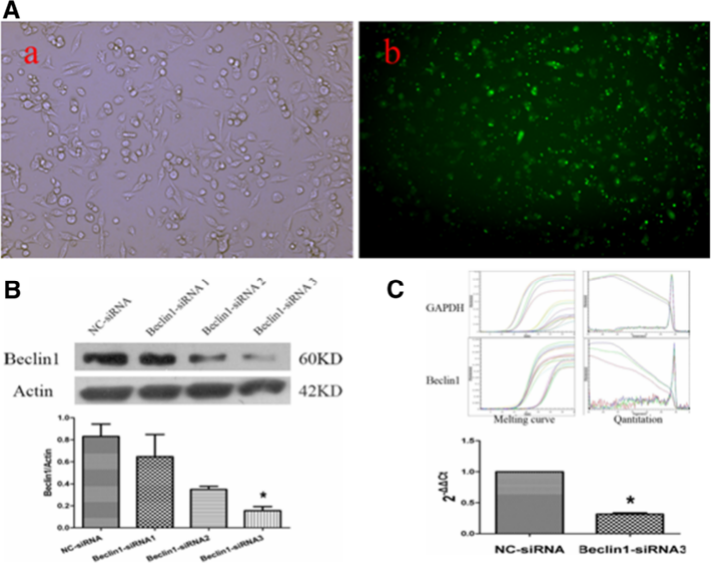
**Detection of Beclin1 expression in Beclin1-siRNA transfected Miapaca2 cells. A**. Miapaca2 cells transfected with FAM-siRNA for 6 h exhibited green fluorescent signals under an inverted fluorescence microscope. **B**. Beclin1 protein expression analysis through Western blotting. Cytoplasmic protein was extracted from the cells of negative control siRNA (NC-siRNA) transfectant and Beclin-siRNA transfectant. The results are representative of at least three independent experiments. Beclin-siRNA3 was found the preferable sequence. **p < 0.01 vs NC-siRNA group. The Student’s *t*-test was used to analyze the data. **C**. Beclin1 mRNA expression analysis through real-time PCR, GAPDH was used to ensure equal loading. ** p < 0.01 vs NC-siRNA group. The Student’s *t*-test was used to analyze the data.

The Miapaca2 cells were transduced with recombinant GFP plasmid and the transfection efficiency was observed by fluorescence microscope. More than 80% of the cells exhibited positive fluorescent signals under an inverted fluorescence microscope (Figure 1A). To observe the effect of Beclin1 inhibition in siRNA transduced Miapaca2 cells, Western blotting was performed to analyze the expression level of Beclin1 protein (Figure 1B). Real-time PCR was also applied to detect Beclin1 mRNA expression recur to comparison of Delta-delta Ct means (Figure 1C). Comparing to NC-siRNA group, less quantity of Beclin1 protein and Beclin1 mRNA were detected in the Beclin1-siRNA transfected Miapaca2 cells. From three Beclin1-siRNA sequences provided, Beclin1-siRNA3 was preferably selected to apply in the following experiment. Further the recombinant plasmids pGPU6/Neo-shBECN1, pGPU6/Neo-shNC and pGPU6/GFP/Neo-shNC were constructed by Shanghai Genepharma Co., Ltd in order to stably transfect cells.

### Beclin1 inhibition increases LC3-II protein expression and formation of autophagosome in Miapaca2 cells

**Figure 2:**
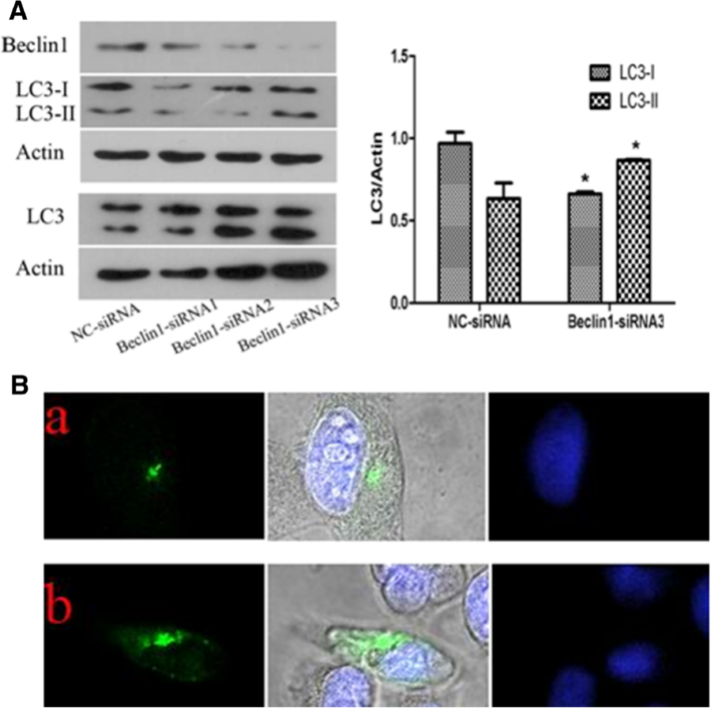
**Inhibition expression of Beclin1 increases LC3-II protein expression and formation of autophagosome in Miapaca2 cells. A**. Beclin1 inhibition increased autophagy in Miapaca2 cancer cells. Western blot analysis of the LC3, Beclin1 expression in Miapaca2 cells transfected with Beclin1-siRNA and NC-siRNA for 48 h. β-Actin was used to ensure equal loading. **B**. Confocal laser scanning microscope for analysis of punctate pattern of LC3 localization in the cells transiently transfected with the GFP-LC3-vector and treated vehicle (Beclin1-siRNA) or control (NC-siRNA) for 48 h. The cells were stained with DAPI. (a:Miapaca2 cells transfected with NC-siRNA; b:Miapaca2 cells transfected with Beclin1-siRNA).

In order to determine whether down-regulation of Beclin1 played a role in regulation of autophagy, we initially determined a change in the expression level of the microtubule-associated protein 1 light chain 3 (LC3)-II, a marker for the presence of autophagosomes, in the Beclin1-siRNA transduced Miapaca2 cells by immunoblot analysis. As shown in Figure 2A, the amount of LC3-II was found increased in Beclin1-siRNA transduced Miapaca2 cells as compared to negative control (NC-siRNA) (p < 0.05), and LC3-I was decreased accordingly (p < 0.05).

In addition, autophagic change of cells expressing GFP-LC3 was also observed. Recruitment of LC3-II to the autophagosomes is characterized by punctate pattern of its localization. The NC-siRNA transduced Miapaca2 cells displayed diffuse and weak LC3-associated green fluorescence. However, the Beclin1-siRNA transduced Miapaca2 cells showed characteristic punctate LC3-associated green fluorescence as shown in Figure 2B (a, b). These results indicated that inhibition expression of Beclin1 was able to promote autophagosome formation in Miapaca2 cells.

### Effect of Beclin1 down-regulation on proliferation of Miapaca2 cells

**Figure 3:**
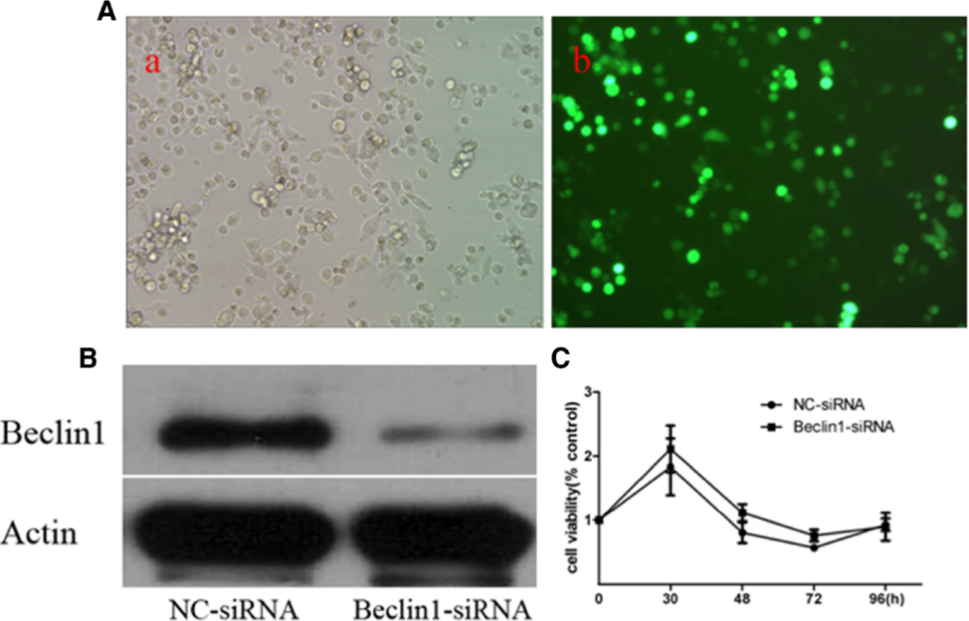
**Effect of down-regulation of Beclin1 on proliferate viability in Miapaca2 cells. A**. Miapaca2 cells transfected with pGPU6/GFP/Neo-shNC for 24 h by Lipofectamine 2000 exhibited green fluorescent signals under an inverted fluorescence microscope. **B**. Miapaca2 cells were stably transfected with pGPU6/Neo-shBECN1 or pGPU6/Neo-shNC for 48 h, then exposed to G418 (400ug/ml) for 18 days. Western blot analyze of the Beclin1 expression. **C**. Small RNA interference of Beclin1 had not obvious influence on survival of Miapaca2 cells in 96 h. Cell viability was determined by the Cell counting Kit-8 assay. The difference was not significant between Beclin1-siRNA group and NC-siRNA group (*p* > 0.05).

Autophagy is recognized as a cytoprotective process against environmental stress and maintaining cellular homeostasis. We sought to determine the effect of autophagy enhancement by Beclin1-siRNA on cell proliferation. First, the Miapaca2 cells transfected with pGPU6/Neo-shBECN1 were selected with G418 (400 μg/ml), then the steady lower-expression of Beclin1 was confirmed by western blot (Figure 3A,B). We examined the proliferation patterns of the transfected cells by CCK8 kit. As shown in Figure 3C, there was not significant variation in the viability of the Beclin1-siRNA transduced Miapaca2 cells compared to that of NC-siRNA cells. The results showed that there was no significant influence of inhibition expression of Beclin1 on the proliferation of Miapaca2 cells as well.

### Effect of Beclin1 inhibition on apoptosis and cell cycle in Miapaca2 cells

**Figure 4:**
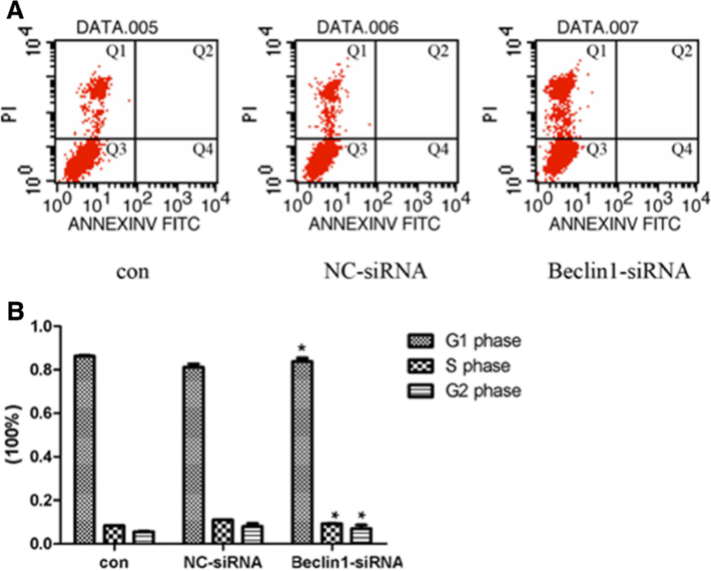
**Effect of down-regulation of Beclin1 on apoptosis and cell cycle in Miapaca2 cells. A**. Apoptosis was determined by flow cytometry for annexin-V-FITC and propidium iodide (PI) dual labeling. Cytograms of annexin-V-FITC binding (abscissa) versus PI uptake (ordinate) show three distinct populations: (1) viable cells in gate Q3; (2) early apoptotic cells in gate Q4; and (3) cells that have lost membrane integrity as a result of very late apoptosis in gate Q2. Percentage of apoptotic cells is gate Q4 plus gate Q2. The apoptotic rate was similar in Beclin1-siRNA transfection Miapaca2 cells (0.01 ± 0.00)% compared with (0.00 ± 0.00)% of NC-siRNA. **B**. Cell cycle was also determined by flow cytometry. The percentage of Beclin1-siRNA transfected Miapaca2 cells in the G_2_ phase was (7.00 ± 2.82)% compared with (7.92 ± 2.56)% of NC-siRNA; (83.80 ± 2.60)% compared with (81.15 ± 2.75)% in the G_1_ phase, and in the S phase was (9.20 ± 0.22)%compared to (10.93 ± 0.19)% accordingly (*p* < 0.01).

In order to observe the effect of autophagy enhancement by Beclin1-siRNA on apoptosis in human pancreatic cancer Miapaca2 cells, the cell apoptosis was stained with Annexin V-FITC and determined by flow cytometry. The results showed that there was no obvious variation of the Annexin V expression in Beclin1-siRNA transduced cells compared to that of NC-siRNA transduced cells (Figure 4A). That indicated the autophagy reinforced by down-regulation of Beclin1 had less influence on the apoptosis of Miapaca2 cells.

In addition, the effect of down-regulation of Beclin1 on Miapaca2 cells cycle was observed. As shown in Figure 4B, the percentage of Beclin1-siRNA transduced Miapaca2 cells in the G_2_ phase was (7.00 ± 2.82)% compared with (7.92 ± 2.56)% of the NC-siRNA cells, and the percentage of the S phase was (9.20 ± 0.22)% compared with (10.93 ± 0.19)% accordingly (*p* < 0.01). These results indicated that autophagy reinforced by down-regulation of Beclin1 had a significant influence on cell cycle arrest of Miapaca2 cells.

### Down-regulation of Beclin1 decreases the cytotoxic sensitivity of Miapaca2 cells to gemcitabine

**Figure 5:**
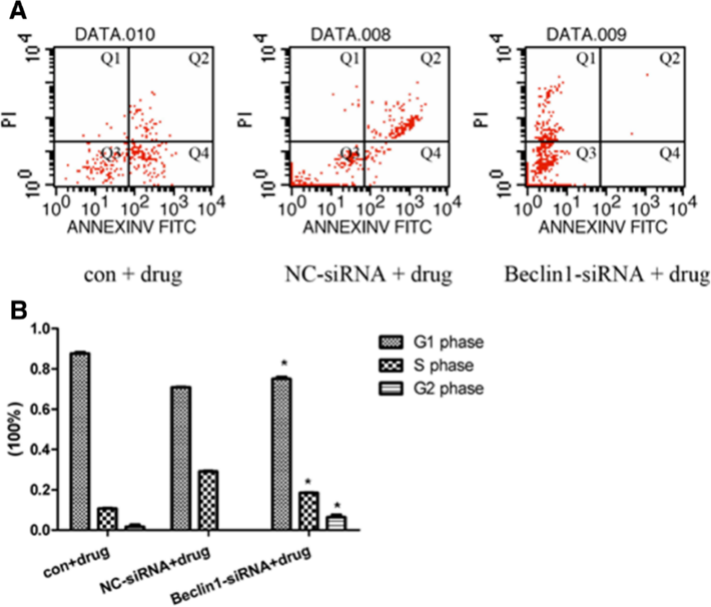
**Down-regulation of Beclin1 decreases the cytotoxic sensitivity of Miapaca2 cells to Gemcitabine. A**. After transfection of vector for 24 h then 24 h of drug incubation in Miapaca2 cells, majority cells went to death. Apoptosis was determined by flow cytometry. The apoptotic rate was significantly decreased to (0.06 ± 0.00)% compared with (29.13 ± 0.47)% of non- drug-treated cells (*p* < 0.01). **B**. Cell cycle was analyzed by flow cytometry. The percentage of Miapaca2 cells treated with Beclin1-siRNA plus drug in the G_2_ phase was (6.46 ± 1.91)% compared with (0.01 ± 0.00)% of NC-control cells; (75.07 ± 1.63)% compared with (71.00 ± 0.45)% in the G_1_ phase, and in the S phase was (18.48 ± 0.28)% compared to (29.00 ± 0.45)% accordingly (*p* < 0.01).

To confirm the effect of autophagy reinforced by Beclin1 inhibition on the cytotoxic sensitivity of Miapaca2 cells, the apoptosis and cell cycle of cells treated with Gemcitabine were analyzed. Gemcitabine alone or plus Beclin1-siRNA induced apoptosis which was determined Annexin V-FITC staining. The results suggested that apoptosis was significantly decreased in Miapaca2 cells treated with Gemcitabine plus Beclin1-siRNA than that of drugs alone (Figure 5A).

Accordingly, Gemcitabine alone or drugs plus Beclin1-siRNA treated cells were analyzed for changes of cell cycle by flow cytometry. As shown in Figure 5B, the percentage of Beclin1-siRNA combining with Gemcitabine treated cells in G_2_ phase was significantly increased to (6.46 ± 1.91)% in compared with (0.01 ± 0.00)% of Gemcitabine alone treated cells, while the percentage of Beclin1-siRNA combining with Gemcitabine treated cells in the S phase was significantly decreased to (18.48 ± 0.28)% compared to (29.00 ± 0.45)% of the drugs alone treated cells (*p* < 0.01), which indicated that Beclin1 inhibition affected cell cycle induced by Gemcitabine and arrested in G_1_–S phase. These results demonstrated that down-regulation of Beclin1 was disadvantageous to survival inhibition of Miapaca2 cells induced by chemotherapeutic anti-cancer drugs Gemcitabine.

## Discussion

Autophagy is a physiologic process that plays an important role in the turnover of cellular proteins and other macromolecules to maintaining cellular homeostasis. There are nearly 30 autophagy-related proteins (ATGs) in mammals, and these have been identified based on investigations in yeast [1]. ATGs, through their roles in signaling pathways, maintain normal physiological levels of autophagy. The autophagy process occurs sequentially in phases: induction, phagophore formation, autophagosome elongation, autolysome formation, and then degradation. During the autophagosome elongation phase, two conjugation systems are indispensable for the formation of the autophagosome. The first one is Atg5-Atg12 conjugates, which are localized to phagophores and dissociate after mature autophagosomes are formed [11]. Atg5-Atg12 conjugation is regulated by the activity of the class III PI-3-kinase, Vps34 [12]. Vps34 activity is positively regulated by the mammalian orthologue of yeast Atg6, Beclin1 [13]. The second is modification involves conjugation of microtubule associated protein 1 light chain 3 (MAP–LC3/Atg8/LC3) to phosphatidylethanolamine (PE). LC3 (cytosolic) is cleaved at its C terminus by Atg4 to form LC3-I. LC3-I is covalently conjugated to PE to form LC3-II. LC3-II (membrane associated) is specifically targeted to Atg5–Atg12-associated, expanded phagophores and remains associated with autophagosomes even after fusion with lysosomes, after which LC3-II can be delipidated and recycled. LC3 is the only known protein that specifically associates with autophagosomes and not with other vesicular structures. Thus, LC3-II levels correlate with autophagosome number, which can also be assessed by scoring LC3-positive vesicles [14]. From above, we have known that Beclin1 mediates the accumulation of ATGs such as Atg5-Atg12 conjugation system and Atg8/LC3 conjugation system locating in pre-autophagosomal structures (PAS), which is essential for the formation of the autophagic vesicle and performs a key role in triggering autophagy and its applications in phagophore formation.

Many observations have confirmed that expression of Beclin1 regulates coincidentally autophagy process. Beclin1 over-expression consistently increased autophagosome numbers [15]. Conversely, Beclin1-deficient mice show a pronounced loss of autophagic vacuole formation.Homozygous Beclin1 null mice die during early embryogenesis and heterozygous Beclin1+/−mice show a high incidence of spontaneous tumors [16]. Similarly, the knockdown of Beclin1 inhibits autophagy and sensitizes cells to starvation-induced cell death [17]. However, the expression and roles of Beclin1 may be different in tumor cells. In our studies, the results were interestingly showed that quantity of LC3-II protein and autophagosome synthesis were significantly increased after Beclin1 had been inhibited through small interfering RNA against beclin1, it meant that Beclin1 inhibition could promote autophagy in human pancreatic cancer cells Miapaca2. The probable mechanisms are assumed that there could be an undiscovered important protein regulating the autophagy process, a possible negative feedback or two-way regulation mechanism played a role when expression of Beclin1 was inhibited. The activity of the protein was associated with Beclin1, meanwhile, it should induce accumulation of Atg5-Atg12 conjugation system and Atg8/LC3 conjugation system in pre-autophagosomal structures, accordingly promote autophagy process. The probable mechanism interests us extremely and that will be our further studies later on. In addition, Beclin1 is part of the type III PI3 kinase complex that is required for the formation of the autophagic vesicle and interacts with Bcl-2. It also interacts with the other major anti-apoptotic Bcl family protein (Bcl-xL). Autophagy can be regulated by this interaction. Inhibiting apoptosis by binding to and interfering with the action of the pro-apoptotic proteins, Bax and Bak, Bcl-2/Bcl-xL also inhibits autophagy by binding with Beclin1. On the basis of our previous results and the current studies, we conjecture the undiscovered important protein regulating the autophagy process could interact with apoptosis/Bcl-2 or Bcl-xL. Besides, the role of autophagy could depend on the type of tumor, the stage of tumorigenesis, and the nature and extent of the insult in tumor cells. The precise and complicated mechanisms underlying the autophagy process are still ambiguous and the further researches are essential to determine.

Recently, a large number of studies on the interconnection between Beclin1, autophagy and tumorigenesis have been analyzed. Numerous observations have demonstrated that Beclin1 has been implicated in tumorigenesis, and plays a role in cellular proliferation. A study also found that the protein level of Beclin1 is lower in cervical cancer tissues than in normal tissues, and is closely related to pelvic lymph node metastases and histologic tumor grade [18]. Heterozygous disruption of Beclin1 in mice results in reduced autophagy, increased cellular proliferation, and spontaneous malignancies [16]. But the molecular mechanism underlying this relationship is still obscure. Pancreatic adenocarcinoma is one of the most lethal human malignancies, it doesn’t respond well to traditional chemotherapy and radiotherapy, leaving little effective treatment for advanced pancreatic cancer cases. The precise complicated mechanisms of insensitivity to therapy are still uncertain completely. Gemcitabine is a widely used chemotherapeutic anti-cancer drug that is effective in the treatment or controlling of a variety of human cancers including pancreatic cancer. Its action is cell phase specific (S-phase) and, under certain circumstances, block the progression of cells through the G1/S-phase boundary. The cytotoxic actions of Gemcitabin *in vitro* are concentration and time dependent. Its cytotoxic effect is due to DNA synthesis inhibition leading to induce apoptosis. In our studies, we proceeded to dissect the contributions of autophagy to the survival of Miapaca2 cells during Gemcitabine-induced apoptosis. The findings suggested down-regulation of Beclin1 increased autophagy, and accordingly inhibited Gemcitabine-induced cell apoptosis in Miapaca2 cells. In addition, that stimulated the progression of cells through the S/G2 phase boundary. Gemcitabine-treated Miapaca2 cells in the G2 phase was significantly increased. According to the present results, we deduced the conclusions that autophagy participated apoptosis process and decreased apoptotic response induced by anticancer therapy in Miapaca2 cells, meanwhile it interfered cell cycle distribution of Miapaca2 cells treated with Gemcitabine, therapeutical effect of Gemcitabine was weakened on Miapaca2 cells in which the expression of Beclin1 was inhibited. Accidentally, an interesting result as Figure 5B shown was found in our experiment, the massive 3-fold increase in cells in the S-Phase of the cell cycle after treatment with non-specific siRNA and gemcitabine. That was departed from the effect of blocking the progression of cells through the G1/S-phase boundary of Gemcitabine. We conjecture that treatment with non-specific siRNA could obstruct the action of Gemcitabine in cell phase specific. The undiscovered mechanism perplexed us and it is necessary to research intensively. A study demonstrated that Beclin1 was not only necessary for the formation of the autophagic vesicle, but also interacted with Bcl-2, an anti-apoptotic protein. And it also interacted with the other major anti-apoptotic Bcl family protein (Bcl-xL) which demonstrated that autophagy could be regulated by this interaction. In addition to inhibiting apoptosis by binding to and interfering with the action of the pro-apoptotic proteins, Bax and Bak, Bcl-2/Bcl-xL also inhibits autophagy by binding with Beclin1[19].

Newly published work by Saleem A *et al.* showed that therapeutic inhibition of autophagy with hydroxychloroquine (HCQ) increased cytotoxicity of small molecule BH3 domain mimetics such as ABT-737 both *in vitro* and *in vivo*[20]. All these results demonstrated potential information that there are possibly precise complicated mechanisms and close correlation between autophagy and the apoptosis in cancer cells. Further research is necessary for tumor cells to anticancer therapy.

## Conclusion

In conclusion, our data indicated Beclin1 inhibition promoted autophagy in human pancreatic cancer cells Miapaca2. It also demonstrated that Beclin1 not only adjusted autophagy process, but also played an important role in the regulation of apoptosis. Beclin1 inhibition could inhibit apoptosis signaling induced by Gemcitabine in Miapaca2 cells. It will be helpful for human to adopt anticancer therapy if the complicated mechanisms could be discoverd in cancer cells.

## Abbreviations

LC3: microtubule-associated protein 1 Light Chain 3.

## Competing interests

The authors declare that they have no competing interests.

## Authors’ contributions

XL carried out most of the experiments and drafted the manuscript. JY and YY participated in the gene knockdown by siRNA and the quantitative RT-PCR assay. FX participated in the cell cycle analysis and the apoptosis assay. XG,HW,LW and JY designed the project, supervised the experiments. All authors read and approved the manuscript.
